# Quantitative Trait Loci Mapping of Heading Date in Wheat under Phosphorus Stress Conditions

**DOI:** 10.3390/genes15091150

**Published:** 2024-08-31

**Authors:** Bin Yang, Ling Qiao, Xingwei Zheng, Jun Zheng, Bangbang Wu, Xiaohua Li, Jiajia Zhao

**Affiliations:** Institute of Wheat Research, Shanxi Agricultural University, Linfen 041000, China; sxxmsyb83@126.com (B.Y.); qiaolingsmile@163.com (L.Q.); smilezxw@126.com (X.Z.); sxnkyzj@126.com (J.Z.); bangbang_wu@126.com (B.W.); lixiaohualff@163.com (X.L.)

**Keywords:** wheat, heading date, phosphorus stress, quantitative trait loci (QTL), molecular markers, candidate genes, wheat breeding, stress tolerance

## Abstract

Wheat (*Triticum aestivum* L.) is a crucial cereal crop, contributing around 20% of global caloric intake. However, challenges such as diminishing arable land, water shortages, and climate change threaten wheat production, making yield enhancement crucial for global food security. The heading date (HD) is a critical factor influencing wheat’s growth cycle, harvest timing, climate adaptability, and yield. Understanding the genetic determinants of HD is essential for developing high-yield and stable wheat varieties. This study used a doubled haploid (DH) population from a cross between Jinmai 47 and Jinmai 84. QTL analysis of HD was performed under three phosphorus (P) treatments (low, medium, and normal) across six environments, using Wheat15K high-density SNP technology. The study identified 39 QTLs for HD, distributed across ten chromosomes, accounting for 2.39% to 29.52% of the phenotypic variance. Notably, five stable and major QTLs (*Qhd.saw-3A.7*, *Qhd.saw-3A.8*, *Qhd.saw-3A.9*, *Qhd.saw-4A.4*, and *Qhd.saw-4D.3*) were consistently detected across varying P conditions. The additive effects of these major QTLs showed that favorable alleles significantly delayed HD. There was a clear trend of increasing HD delay as the number of favorable alleles increased. Among them, *Qhd.saw-3A.8*, *Qhd.saw-3A.9*, and *Qhd.saw-4D.3* were identified as novel QTLs with no prior reports of HD QTLs/genes in their respective intervals. Candidate gene analysis highlighted seven highly expressed genes related to Ca^2+^ transport, hormone signaling, glycosylation, and zinc finger proteins, likely involved in HD regulation. This research elucidates the genetic basis of wheat HD under P stress, providing critical insights for breeding high-yield, stable wheat varieties suited to low-P environments.

## 1. Introduction

Wheat is one of the world’s most essential cereal crops [1]. By 2050, the global population is projected to exceed nine billion, leading to a 60% increase in wheat demand [2]. However, wheat production faces significant challenges due to reduced arable land, water scarcity, and climate change [3]. Thus, improving wheat yield is crucial for ensuring global food security.

The heading date (HD) refers to the time required for more than half of the wheat plants to head after sowing. The HD affects not only the growth cycle and harvest timing of wheat but also its adaptability to different climatic conditions and its final yield [4,5]. An appropriate HD helps wheat avoid adverse climatic conditions, such as drought, frost, and high temperatures, thereby enhancing yield and quality. Therefore, understanding the genetic mechanisms underlying HD is critical for breeding high-yielding, stable, and environmentally adaptable wheat varieties.

The HD of wheat is a complex quantitative trait regulated by the interaction of multiple genes and environmental factors [6]. In recent years, advancements in molecular marker technology and genome-wide association studies (GWASs) have led to the identification of over 200 QTLs (quantitative trait loci) associated with wheat HD [7,8,9,10]. It has been found that wheat HD is primarily controlled by three categories of genes: vernalization (*VRN*) response, photoperiod (*PPD*) response, and earliness per se (*EPS*). Additionally, several key genes related to HD have been cloned.

The *VRN* gene promotes flowering through prolonged exposure to low temperatures and is primarily regulated by the *VRN1* [11], *VRN2* [12], *VRN3* [13], and *VRN-D4* [14] genes. The *VRN1* gene, which promotes flowering after vernalization, is located on the 5A, 5B, and 5D chromosomes, designated as *VRN-A1*, *VRN-B1*, and *VRN-D1*, respectively [11]. Allelic variations in *VRN1* are the main source of genetic variation in wheat’s vernalization requirement and are associated with differences in heading dates among winter wheat varieties from different geographical regions [15]. The *VRN2* gene, located on the 4B and 5A chromosomes, encodes a zinc finger-CCT domain transcription factor that inhibits flowering in cereal crops. Under unvernalized conditions, *VRN2* is highly expressed, suppressing flowering; however, during vernalization, *VRN2* expression significantly decreases, thereby lifting the inhibition on flowering [12]. The *VRN3* gene, homologous to the Arabidopsis *FLOWERING LOCUS T* (*FT*) gene, is located on the 7A, 7B, and 7D chromosomes. Its gene product acts as a mobile signal protein that moves from leaves to the shoot apical meristem to accelerate flowering [13]. *VRN3* expression is regulated by *VRN1*, with upregulation of *VRN3* helping to relieve flowering inhibition and promote wheat flowering. The *VRN-D4* gene is located in the centromeric region of the 5D chromosome and is found only in the D genome. *VRN-D4* interacts significantly with other vernalization genes, such as *VRN-A1*, *VRN-B1*, *VRN-D1*, and *VRN-B3*, and is involved in the vernalization pathway [14].

*PPD* is another crucial environmental factor affecting the HD of wheat. In wheat, the primary photoperiod response genes include *Ppd-D1*, *Ppd-B1*, *Ppd-A1*, and *Ppd-B2*, located on chromosomes 2D, 2B, 2A, and 7BS, respectively. A mutation in the *Ppd-D1* gene (*Ppd-D1a*) converts wheat from a long-day plant to a photoperiod-insensitive plant, promoting flowering under both short-day and long-day conditions by activating the *FT* gene [16]. Mutations in the *Ppd-B1* and *Ppd-A1* genes (*Ppd-B1a* and *Ppd-A1a*) also confer photoperiod insensitivity to wheat, enabling it to flower more quickly under varying photoperiod conditions [17,18]. Additionally, the *Ppd-B2* gene exhibits strong expression under long-day conditions, accelerating flowering and being associated with increased grain protein content [19].

*EPS* genes regulate the fine-tuning of flowering time in wheat, independent of *VRN* and *PPD*. The *EPS-Am1* gene, located on the 1AmL chromosome arm of diploid wheat, is temperature-sensitive and affects the duration of different developmental stages and the number of spikelets [20]. Studies have suggested that *EPS-Am1* is a candidate gene for the circadian regulator *ELF3*, with amino acid differences in the ELF3 protein associated with variations in flowering time and spikelet number [21]. In hexaploid wheat, the *EPS-D1* gene is located on chromosome 1D and the *EPS-3A* gene on chromosome 3A, both significantly influencing the duration of various growth stages and the number of spikelets during the wheat lifecycle [22].

Although many QTLs and genes regulating HD in wheat have been identified, their specific effects under various environmental conditions, especially P stress, remain unclear. Therefore, it is essential to utilize high-density molecular marker technology to detect QTLs for HD across diverse environments. Identifying major QTLs consistently detected in multiple environments provides a crucial foundation for subsequent positional cloning and functional gene studies [23].

P is an essential element for plant growth and development, playing a crucial role in energy transfer, signal transduction, and metabolic regulation. However, the available P content in global soils is generally insufficient, making P stress one of the primary factors limiting wheat production [24]. P stress not only affects wheat growth and development but also disrupts physiological metabolism, thereby influencing the HD and final yield [25]. In the North China Plain, a major wheat-producing region in China, most soils contain less than 10 mg/kg of available P [26]. Therefore, studying the genetic mechanisms of wheat HD under P stress is of great significance for improving wheat adaptability and yield in low-P environments.

This study aims to investigate the genetic basis of HD regulation in wheat under P stress conditions. We hypothesize that specific QTLs associated with HD can be consistently identified across different P environments, providing critical insights into the mechanisms governing HD under nutrient stress. The primary objective is to identify and characterize major and stable QTLs that contribute to HD regulation under varying P conditions and to explore potential candidate genes associated with these QTLs. These findings are expected to enhance the understanding of the impact of P stress on wheat HD but also offer theoretical and practical guidance for breeding high-yield, stable wheat varieties adaptable to low-P environments.

## 2. Materials and Methods

### 2.1. Plant Materials and Plot Design

The experimental materials used in this study included a DH population of 201 lines derived from a cross between Jinmai 47 and Jinmai 84. Both Jinmai 47 and Jinmai 84 were developed by the Cotton Research Institute of Shanxi Agricultural University. Jinmai 47 is characterized by early heading and strong drought resistance, while Jinmai 84 is a late-heading, high-yield variety suitable for irrigated conditions.

During the 2021–2022 period, the DH population and parent lines were cultivated at the Hancun Experimental Station, part of the Wheat Research Institute at Shanxi Agricultural University, Shanxi Province, China. The station is situated at an elevation of 459.00 m (111°34′36″ E, 36°8′43″ N) within the North China Plain, which features a typical temperate continental semi-arid climate. The area receives an average annual rainfall of 457.71 mm and maintains an average annual temperature of 13.08 °C [27].

The experiment incorporated three P treatments: low P (E1 and E2), medium P (E3 and E4), and normal P (E5 and E6). P was applied at rates of 196 kg/ha of P_2_O_5_ for normal P, 84 kg/ha for medium P, and no P fertilizer for low P [28]. All plots were uniformly treated with 168.75 kg/ha of urea and 49.17 kg/ha of potassium chloride. The data of nutritional analysis of the soil are shown in Table S1. The design was a completely randomized block with three replications per treatment. Each plot consisted of two rows, each 1.5 m in length, spaced 0.3 m apart, and sown with 21 seeds per row. Irrigation was administered at the pre-winter, jointing, and flowering stages, with each session delivering 700 cubic meters per hectare. Other agronomic practices followed local standards.

### 2.2. Phenotypic Evaluation and Data Analysis

The heading time for each plant was recorded when two-thirds of the spikes had emerged [7]. To ensure consistency, measurements were taken at the same time each day. Analysis of variance (ANOVA), Pearson correlation analysis, and Student’s *t*-test (*p* < 0.05) were performed on phenotype values across different environments using SPSS 21.0 software (SPSS, Chicago, IL, USA) [28]. Tukey’s HSD test was subsequently used to calculate the effects of specific factors on the phenotypic traits. SAS (SAS Institute, Cary, NC, USA, https://www.sas.com/en_in/home.html) was used to calculate the best linear unbiased prediction (BLUP) and broad-sense heritability (*H*^2^) for HD in various environments [29]. Population phenotype frequency distributions and figures were generated using Origin2018 software (OriginLab, Northampton, MA, USA).

### 2.3. Genetic Linkage Map Construction and QTL Mapping

DNA from fresh leaves of each DH line and parent was extracted using a modified cetyl trimethylammonium bromide (CTAB) method [30]. After confirming DNA integrity, concentration, and purity, the DH population was genotyped with the Illumina Infinium Wheat15K SNP chip. Redundant markers were removed using the BIN program, and linkage maps were constructed with QTL IciMapping 4.1 and visualized using JoinMap 4.0 [31].

QTL mapping was conducted with WinQTLCart 2.5 (https://brcwebportal.cos.ncsu.edu/qtlcart/WQTLCart.htm, accessed on 15 April 2024) using the composite interval mapping method. An LOD score threshold of 2.5 was set to identify significant QTLs. QTLs explaining more than 10% of the phenotypic variance and detected in at least three environments (including BLUP) were considered major and stable QTLs. QTLs less than 1 cM apart or sharing common flanking markers were regarded as a single locus and named according to McCouch et al. [32].

### 2.4. Identification of Candidate Genes

Candidate genes within the newly identified major QTL intervals were identified using the Interval Tool platform on WheatOmics 1.0 website (http://wheatomics.sdau.edu.cn, accessed on 25 May 2024) [33]. Functional annotations of previously reported genes were considered to preliminarily screen for genes associated with HD. Expression data for these genes were obtained from the expVIP database (http://www.wheat-expression.com/, accessed on 25 May 2024), and differential expression analysis was conducted for tissues related to HD, such as coleoptile, leaf, shoots, and second leaf. This analysis ultimately identified potential candidate genes.

## 3. Results

### 3.1. Phenotypic Evaluation

**Figure 1:**
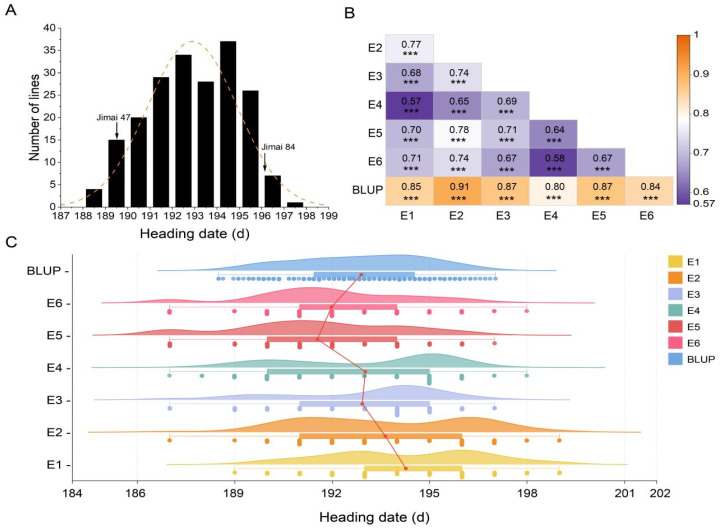
Phenotypic analysis of heading date (HD) in the DH population under different phosphorus (P) levels. (**A**) Distribution of HD in the DH population across varying P levels. Arrows indicate the positions of the parental lines Jinmai 47 and Jinmai 84. The data presented are based on the best linear unbiased prediction (BLUP) values. (**B**) Pearson correlation coefficients of HD between different P levels (E1 to E6) and BLUP values. Asterisks denote significance levels: *** *p* < 0.001. (**C**) Density distribution of HD in the DH population under different P treatments.

The results indicated that the HD of the DH population ranged from 188 to 197 days, with most lines having an HD between 193 and 195 days (Table 1, Figure 1A). The parents, Jinmai 47 and Jinmai 84, exhibited HD of approximately 189 days and 196 days, respectively, with significant differences (*p* < 0.01) observed between the two parents across all environments (Table 1, Figure 1A). Correlation analysis showed significant correlations (*p* < 0.001) of HD among different environments, with correlation coefficients ranging from 0.57 to 0.91 (Figure 1B). Density distribution analysis revealed that low P stress (E1 and E2) significantly extended the HD of the DH population, whereas HD was relatively shorter under normal and medium P conditions (E3, E4, E5, and E6) (Figure 1C), indicating that low P stress significantly affects wheat HD.

The *H*^2^ of HD in the DH population was greater than 0.80, indicating high genetic stability (Table 1). The skewness and kurtosis values of the HD data were mostly less than 1.0, showing a continuous normal distribution across environments. This suggests polygenic control and suitability for QTL analysis.

### 3.2. Genetic Linkage Map Construction

**Figure 2:**
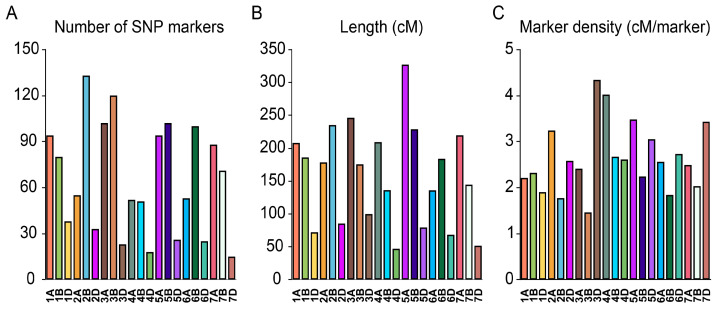
Genomic distribution of SNP markers, map length, and marker density across chromosomes. (**A**) Number of SNP markers on each chromosome. (**B**) Length of each chromosome in centiMorgans (cM). (**C**) Marker density (cM/marker) for each chromosome in the wheat genome.

In this study, the DH population derived from the cross between Jinmai 47 and Jinmai 84 was genotyped using the Illumina Infinium Wheat15K SNP chip (MOL-BREEDING Company, Shijiazhuang, China). After filtering and selection, a total of 1373 SNP markers were obtained and used to construct the genetic linkage map. These markers were distributed across the 21 wheat chromosomes, covering a total length of 3316.06 cM, with an average marker density of 2.42 cM per marker (Figure 2A–C).

The number of SNP markers, chromosome lengths, and marker densities varied across the chromosomes. Chromosome 2B contained the highest number of SNP markers (133), while chromosome 7D had the fewest (15) (Figure 2A). Chromosome 5A had the greatest coverage length at 327.02 cM, whereas chromosome 4D had the smallest at 46.93 cM (Figure 2B). The highest marker density was observed on chromosome 3B, with 1.46 cM per marker, and the lowest on chromosome 3D, with 4.34 cM per marker (Figure 2C). Overall, the SNP markers were evenly distributed across the chromosomes, providing comprehensive genome coverage and a robust foundation for subsequent QTL mapping.

### 3.3. QTL Mapping for Heading Date under Different Phosphorus Conditions

A total of 39 QTLs for HD were identified under different P levels (low, medium, normal) as well as in the BLUP analysis. These QTLs were distributed across 10 chromosomes, including 1B, 2B, 3A, 3B, 4A, 4B, 4D, 5A, 5D, and 7B (Table 2), and explained 2.39% to 29.52% of the phenotypic variance (PVE), with LOD scores ranging from 2.52 to 22.14. Among these QTLs, 16 had favorable alleles derived from the female parent, Jinmai 47, while 23 had favorable alleles from the male parent, Jinmai 84 (Table 2).

Five stable and major QTLs were detected, including *Qhd.saw-4D.3* on chromosome 4D, which was identified in E1, E2, E3, E4, E5, and BLUP analyses, with LOD scores ranging from 4.46 to 22.14 and explaining 8.35% to 29.52% of the PVE. The additive effect ranged from −1.32 to −0.97 days. Three stable and major QTLs located on chromosome 3A were identified as *Qhd.saw-3A.7*, *Qhd.saw-3A.8*, and *Qhd.saw-3A.9*. *Qhd.saw-3A.8* was detected in all environments and in BLUP, with LOD scores ranging from 5.51 to 14.03, explaining 7.80% to 15.83% of the PVE, and with an additive effect ranging from −0.94 to −0.72 days. *Qhd.saw-3A.7* and *Qhd.saw-3A.9* were identified in four (E2, E4, E5, and BLUP) and six environments (E1, E2, E4, E5, E6, and BLUP), respectively, with the highest PVE explained reaching 14.65%. Another stable QTL, *Qhd.saw-4A.4*, was detected on chromosome 4A in E1, E2, E5, E6, and BLUP, with LOD scores ranging from 3.08 to 8.38, explaining 4.73% to 12.04% of the PVE, and with an additive effect ranging from 0.53 to 0.84 days (Table 2).

Additionally, some QTLs were identified specifically under low P conditions. For instance, *Qhd.saw-1B.2* and *Qhd.saw-4D.2* were only detected in E2, indicating their specific response to low P stress (Table 2).

### 3.4. Additive Effects Analysis of the Five Major QTLs

**Figure 3:**
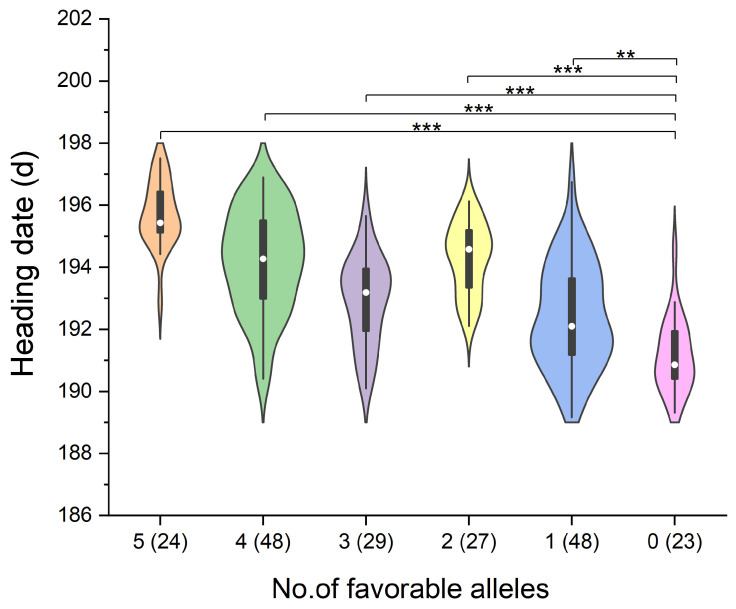
Relationship between the number of favorable alleles of major QTLs and heading date (HD) in wheat (BLUP data). Violin plots show the distribution of HD values for different numbers of favorable alleles. The numbers in parentheses indicate the number of lines within each group. Statistical significance is indicated by asterisks (** *p* < 0.01, *** *p* < 0.001).

The analysis of the additive effects of the five stable and major QTLs (*Qhd.saw-3A.7*, *Qhd.saw-3A.8*, *Qhd.saw-3A.9*, *Qhd.saw-4A.4*, and *Qhd.saw-4D.3*) revealed that the presence of favorable alleles at these major QTLs significantly delayed the HD of wheat. Moreover, an increasing number of favorable alleles was associated with a progressively greater delay in HD (Table 3, Figure 3). Specifically, the average HD for the 24 lines carrying all five favorable alleles was 195.72 days, which was 4.58 days longer than the average HD of 191.14 days observed in the 23 lines with no favorable alleles, representing a delay of 2.40% (Table 3).

### 3.5. Prediction and Analysis of Candidate Genes for Qhd.saw-3A.8, Qhd.saw-3A.9, and Qhd.saw-4D.3

Previous studies identified 12, 7, and 1 QTLs regulating wheat HD on chromosomes 3A, 4A, and 4D, respectively, using various genetic populations (Table 4). By referencing the Chinese Spring genome sequence v1.0 database, it was found that *Qhd.saw-3A.7* (609.429–623.405 Mb) partially overlaps with the HD marker interval (569.43–649.03 Mb) reported by Mohler et al. [34]. *Qhd.saw-4A.4* (676.486–638.262 Mb) coincides with the HD marker interval (669.58 Mb) reported by Fan et al. [35]. However, *Qhd.saw-3A.8*, *Qhd.saw-3A.9*, and *Qhd.saw-4D.3*, located at 650.835–655.885 Mb, 681.755–688.622 Mb on chromosome 3A, and 28.547–15.772 Mb on chromosome 4D, respectively, have not been associated with previously reported QTLs or genes related to HD (Table 4). This suggests that *Qhd.saw-3A.8*, *Qhd.saw-3A.9*, and *Qhd.saw-4D.3* may represent novel major QTLs regulating HD.

**Figure 4:**
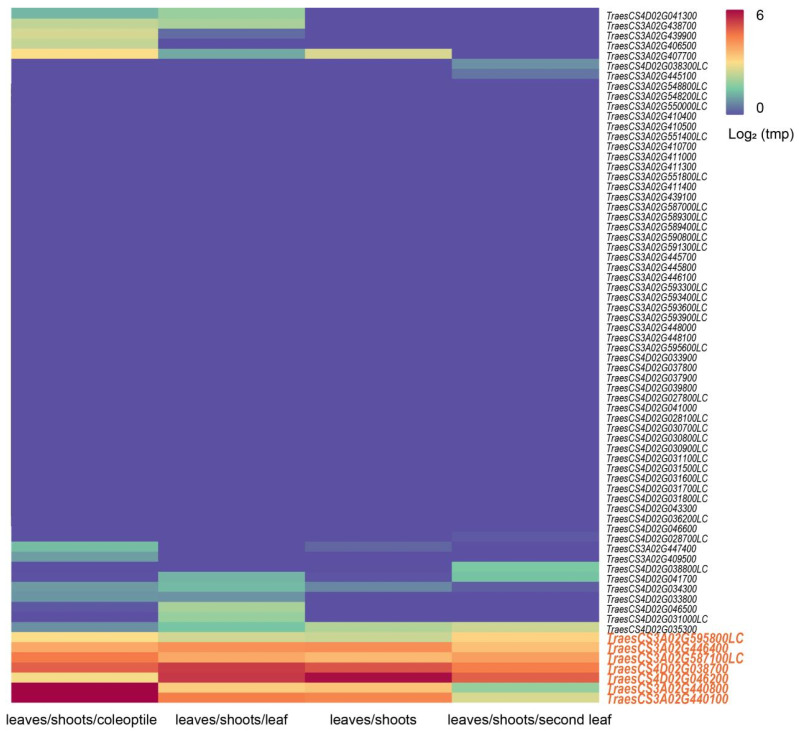
Expression profiles of candidate genes in wheat tissues. A heatmap illustrating the expression levels of candidate genes associated with heading date across various wheat tissues (coleoptile, leaf, shoots, and second leaf). Expression levels are presented on a log2 scale, with colors ranging from low (blue) to high (red) expression.

Functional annotation of genes within the intervals of *Qhd.saw-3A.8*, *Qhd.saw-3A.9*, and *Qhd.saw-4D.3* identified a total of 69 candidate genes associated with HD, including 14 genes in the *Qhd.saw-3A.8* interval, 25 in the *Qhd.saw-3A.9* interval, and 30 in the *Qhd.saw-4D.3* interval (Table S2). Further expression pattern analysis using expVIP platform identified seven highly expressed candidate genes associated with HD, including five on chromosome 3A and two on chromosome 4D (Figure 4). Among these candidate genes, three were found to be homologous to rice genes (Table 5). The candidate gene *TraesCS3A02G595800LC* on chromosome 3A encodes a calcium-dependent lipid-binding protein (CaLB domain); *TraesCS3A02G446400* is associated with an F-box protein containing Kelch repeats; *TraesCS3A02G587100LC*, *TraesCS3A02G440800*, and *TraesCS3A02G440100* encode UDP-glycosyltransferase 84A1 and members of the glycosyltransferase family, respectively. On chromosome 4D, the candidate genes *TraesCS4D02G038700* and *TraesCS4D02G046200* encode a zinc finger family protein and a CONSTANS-like zinc finger protein, respectively. Overall, the functional annotation and expression analysis of these candidate genes provide critical insights into the genetic regulation of HD in wheat, particularly under P stress conditions.

## 4. Discussion

### 4.1. The Impact of Phosphorus Stress on Wheat Heading Date

This study, which examined the HD of a DH population derived from Jinmai 47 × Jinmai 84 under three P levels, revealed that P stress significantly affects wheat HD. Specifically, under low P conditions (E1 and E2), the HD of wheat was notably delayed, whereas it was relatively shorter under normal (E5 and E6) and medium P conditions (E3 and E4) (Table 1). These findings support our hypothesis that P, as a crucial element for plant growth and development, significantly influences wheat’s physiological processes, especially under stress conditions.

P is essential for plant growth and development, playing a key role in energy transfer, signal transduction, and metabolic regulation. When P is limited, plants experience reduced P content, which negatively impacts photosynthesis and respiration, thereby delaying HD [47,48]. This delay is accompanied by decreased synthesis of adenosine triphosphate (ATP) and ribonucleic acid (RNA), imbalances in carbon and nitrogen metabolism, and altered hormone levels, such as increased abscisic acid (ABA) and ethylene, which collectively impact wheat growth and development [25,49]. These physiological changes underscore the critical role of P in maintaining normal growth cycles, particularly in the timing of HD.

Interestingly, under medium P conditions, HD was not significantly extended. This suggests that wheat may have inherent tolerance mechanisms. Under P-limiting conditions, plants activate various adaptive mechanisms to enhance P uptake and utilization efficiency, thereby mitigating the negative effects of P stress [50,51]. Research has shown that plants under P stress increase root length and branching to improve P absorption efficiency [52,53]. These adaptive responses highlight the resilience of wheat and its ability to partially mitigate the adverse effects of P stress, which could explain the less-pronounced delay in HD under medium P conditions. Additionally, P stress induces the expression of genes related to P uptake and utilization, such as phosphate transporter genes (*PHT1*) and acid phosphatase genes (*ACP1*) [54,55]. The high expression of these genes emphasizes their potential role in helping wheat adapt to low-P environments, offering insights into the genetic mechanisms behind this adaptability.

However, in the North China Plain, one of China’s most important wheat-producing regions, the available P content in most soils is generally low, creating a typical P stress environment [26]. This study found that under low P stress (E1 and E2), wheat’s self-protection mechanisms were insufficient to fully mitigate P stress. This led to delayed growth and development, significantly extended HD, and ultimately reduced yield potential. This finding underscores the practical importance of understanding the genetic mechanisms underlying wheat HD under P stress, as it directly impacts agricultural productivity in P-deficient regions.

### 4.2. Comparison of Stable and Major QTLs with Previous Studies

Wheat HD is a complex quantitative trait that is highly influenced by environmental factors. These factors regulate the expression and function of related genes, ultimately determining the growth period and adaptability of wheat. Identifying major QTLs that are stably expressed across various P environments is therefore crucial for understanding the genetic mechanisms underlying HD and for breeding P-efficient wheat varieties through marker-assisted selection.

In this study, thirty-nine QTLs controlling HD were identified under different P environments using a DH population derived from Jinmai 47 × Jinmai 84. Among them, five stable and major QTLs (*Qhd.saw-3A.7*, *Qhd.saw-3A.8*, *Qhd.saw-3A.9*, *Qhd.saw-4A.4*, and *Qhd.saw-4D.3*) were consistently expressed across multiple P environments (Table 2). These QTLs support our hypothesis, indicating that certain genetic loci remain stable across varying environmental conditions, thus providing a reliable genetic basis for HD regulation under P stress. This stability is crucial for developing wheat varieties that can thrive under nutrient stress. 

For example, *Qhd.saw-3A.7*, located between 609.429 Mb and 623.405 Mb on chromosome 3A, overlaps with a QTL (IWA7159–IWA4298) identified by Mohler et al. that was stably expressed across various environments, with an LOD score of 16.8 [34]. In the adjacent region around 625.79 Mb, Griffiths et al. identified nine QTLs related to HD through meta-analysis [42]. *Qhd.saw-4A.4*, located between 638.262 Mb and 676.486 Mb on chromosome 4A, coincides with an HD QTL (AX-95239105–AX-111711476) reported by Fan et al. [35]. Additionally, two HD-related QTLs were identified nearby, around 626.06 Mb and 695.97 Mb [34,46]. The detection of *Qhd.saw-3A.7* and *Qhd.saw-4A.4* in both this study and previous studies suggests that these major QTLs are relatively stable and less influenced by environmental and genetic backgrounds, reinforcing their potential as targets for wheat-breeding programs.

In contrast, no previously reported HD QTLs or genes have been identified within the intervals of *Qhd.saw-3A.8* (650.835–655.885 Mb), *Qhd.saw-3A.9* (681.755–688.622 Mb), and *Qhd.saw-4D.3* (28.547–15.772 Mb), indicating that these may represent novel major QTLs regulating HD. Interestingly, several QTLs and genes related to yield traits have been mapped within these regions. For example, a QTL for thousand kernel weight (TKW), *TKW-IWB26056*, was identified by Sun et al. near 657.61 Mb on chromosome 3A [56], and another TKW-related QTL, *QTKW.caas-3AL*, was identified by Li et al. near 687.59 Mb [38]. On chromosome 4D, between 15.772 Mb and 28.547 Mb, two TKW QTLs, *QTKW-4D-AN* and *QTgw-4D*, were identified [34,57], along with a major chlorophyll QTL, *Qchl.saw-2D.2* [28], and a gene, *TB-D1*, controlling spike architecture in wheat at 18.46 Mb, as reported by Dixon et al. [58]. The discovery of these QTLs not only expands our understanding of the genetic regulation of HD but also suggests a potential pleiotropic effect, linking HD to important yield traits. This connection underscores the importance of these QTLs in developing wheat varieties that are both P-efficient and high yielding.

### 4.3. Candidate Gene Analysis

Functional analysis of the regions associated with three newly identified major QTLs (*Qhd.saw-3A.8*, *Qhd.saw-3A.9*, and *Qhd.saw-4D.3*) led to the identification of seven candidate genes potentially related to HD regulation (Table 5, Figure 4). These genes are involved in various physiological processes, including Ca^2^⁺ transport and signaling, plant hormone signaling, glycosylation reactions, and zinc finger protein function. Further validation of these candidate genes will be crucial for understanding their specific roles in HD regulation under P stress, and could provide valuable insights for wheat-breeding strategies aimed at improving adaptability to low-P environments.

*TraesCS3A02G595800LC* is a key gene encoding a calcium-dependent lipid-binding family protein. Ca^2^⁺ acts as a second messenger in plants, regulating various physiological responses related to plant development [59]. It is involved in the formation of Ca^2^⁺ sensors and the transmission of calcium signals, thereby influencing wheat growth, development, and stress responses [33]. Consequently, it plays a significant role in the regulation of flowering time [60].

*TraesCS3A02G446400*, located on chromosome 3A, encodes an F-box family protein. F-box proteins play crucial roles in plant hormone signaling, light signal transduction, cell division, floral meristem development, and organ formation [61,62]. They influence plant growth and development through the regulation of the ubiquitin–proteasome pathway [63]. By regulating protein degradation, F-box proteins may directly affect flowering time, thereby influencing HD in wheat.

Additionally, three genes are related to glycosyltransferases. Glycosylation, catalyzed by glycosyltransferases, is a necessary modification process for plant cell growth, development, and metabolic balance, playing vital roles in seed germination, growth, flowering, and fruiting [64]. The gene *TraesCS3A02G587100LC* encodes UDP-glycosyltransferase 84A1, which is crucial in the glycosylation process in plants. In Medicago truncatula, the homologous gene MtUGT84A1 plays an important role in jasmonic acid (JA) signaling and anthocyanin accumulation [65]. The genes *TraesCS3A02G440800* and *TraesCS3A02G440100* encode glycosyltransferase family exostosin proteins, which influence cell division and differentiation by regulating molecular signal transduction [66]. In rice, the homologous gene *Os01t0926600-01*, encoding probable glucuronosyltransferase, participates in the synthesis of glucuronoxylan hemicellulose in the secondary cell wall, impacting plant growth and development [67].

On chromosome 4D, two genes, *TraesCS4D02G038700* and *TraesCS4D02G046200*, encode zinc finger family proteins involved in the physiological and biochemical regulation of plant growth and development. Zinc finger-homeodomain transcription factors (ZF-HDs) in wheat can bind to DNA to regulate the expression levels of target genes, thereby participating in wheat growth and development [68]. *TraesCS4D02G046200* encodes a CONSTANS-like zinc finger protein, with its rice homolog *Os03t0711100-01* encoding CONSTANS-like 16. Studies have shown that CONSTANS-like genes play a crucial role in regulating the photoperiod response pathways and flowering time in crops such as wheat and barley [69,70]. These candidate genes represent promising targets for future research aimed at unraveling the complex genetic network governing HD under P stress, ultimately contributing to the development of wheat varieties optimized for growth in low-P soils.

## 5. Conclusions

This study identified thirty-nine QTLs associated with HD in wheat under varying P stress conditions using a DH population derived from Jinmai 47 and Jinmai 84. Among these, five major and stable QTLs (*Qhd.saw-3A.7*, *Qhd.saw-3A.8*, *Qhd.saw-3A.9*, *Qhd.saw-4A.4*, and *Qhd.saw-4D.3*) were consistently detected across different P environments, providing crucial insights into the genetic regulation of HD under P stress. The consistent detection of these QTLs supports our hypothesis, suggesting that these loci are integral to HD regulation across varying environmental conditions. Notably, three novel QTLs (*Qhd.saw-3A.8*, *Qhd.saw-3A.9*, and *Qhd.saw-4D.3*) were identified, which have not been previously reported in association with HD, suggesting their potential as targets for future wheat-breeding programs. Furthermore, candidate gene analysis highlighted seven genes likely involved in HD regulation, related to Ca^2^⁺ transport, hormone signaling, glycosylation, and zinc finger proteins. These findings enhance our understanding of the genetic mechanisms underlying wheat’s response to P stress and provide valuable markers for the development of high-yield, stable wheat varieties suited to low-P environments. Future research should focus on validating these QTLs and candidate genes across diverse environmental conditions and exploring their functional roles to fully harness their potential in wheat breeding.

## Figures and Tables

**Table 1:** Statistical analysis of heading date (HD) for parents and the DH population under different phosphorus environments.

	Parent		DH Population					
	Jinmai 47	Jinmai 84	Mean	SD	Range	Kurtosis	Skewness	*H* ^2^
E1	190.33 **	196.67	194.27	2.41	189–199	−0.86	−0.06	0.8171
E2	189.33 **	198.67	193.66	2.68	188–199	−0.93	−0.04	
E3	189.67 **	196.67	192.93	2.48	187–197	−0.42	−0.73	
E4	189.33 **	195.67	193.02	2.58	187–198	−1.27	−0.40	
E5	188.33 **	194.67	191.62	2.54	187–197	−0.61	−0.11	
E6	188.67 **	195.67	192.04	2.38	187–198	−0.09	−0.04	
BLUP	189.53 **	196.09	192.90	2.01	188.50–197.03	−0.82	−0.17	

Note: SD, standard deviation; *H*^2^, broad-sense heritability; and BLUP, best linear unbiased prediction. Significant differences between Jinmai 47 and Jinmai 84 are indicated by asterisks (** *p* < 0.01).

**Table 2:** QTL for heading date (HD) identified in different phosphorus environments.

QTL	Environment	LOD	Add.	*R* ^2^	Left Marker	Right Marker	Interval (cM)	Physical Interval (Mb)
*Qhd.saw-1B.1*	E2\E5\BLUP	2.66–3.21	−0.52–−0.32	2.64–3.82	1B_654087630	1B_643455303	55.9–69.9	654.087–643.455
*Qhd.saw-1B.2*	E2	2.65	−0.48	3.17	1B_454990828	1B_378500287	169.3–177.7	454.990–378.500
*Qhd.saw-2B.1*	E6	2.61	0.45	3.91	2B_122765594	2B_95067270	102.4–108.5	122.765–95.067
*Qhd.saw-2B.2*	E6	2.88	0.47	4.29	2B_72594141	2B_67981922	119.1–123.6	72.594–67.981
*Qhd.saw-3A.1*	E4\E5	3.06–4.81	−0.63–−0.55	4.45–6.74	3A_33447270	3A_60938981	77.7–86.9	33.447–60.938
*Qhd.saw-3A.2*	E5	4.79	−0.63	6.70	3A_69702468	3A_106442355	89.5–93.0	69.702–106.442
*Qhd.saw-3A.3*	E5	6.55	−0.73	8.99	3A_179770248	3A_207453847	98.5–101.0	179.770–207.453
*Qhd.saw-3A.4*	E5	7.55	−0.78	10.25	3A_484578779	3A_477796895	108.5–109.9	484.578–477.796
*Qhd.saw-3A.5*	E4	4.13	−0.63	5.94	3A_492185407	3A_525825022	112.4–115.4	492.185–525.825
*Qhd.saw-3A.6*	E4\E5	3.84–9.24	−0.91–−0.61	5.55–13.88	3A_534326783	3A_597825929	123.5–131.4	534.326–597.825
*Qhd.saw-3A.7*	E2\E4\E5\BLUP	5.37–12.81	−0.92–−0.71	7.61–14.65	3A_609429465	3A_623405134	135.7–139.5	609.429–623.405
*Qhd.saw-3A.8*	E1\E2\E3\E4\E5\E6\BLUP	5.51–14.03	−0.94–−0.72	7.80–15.83	3A_650835555	3A_655885348	142.0–149.4	650.835–655.885
*Qhd.saw-3A.9*	E1\E2\E4\E5\E6\BLUP	5.72–12.85	−0.92–−0.69	8.40–14.97	3A_681755353	3A_688622043	152.8–157.0	681.755–688.622
*Qhd.saw-3A.10*	E5	8.40	−0.89	13.49	3A_688693273	3A_701009953	160.7–167.6	688.693–701.009
*Qhd.saw-3B.1*	BLUP	2.57	0.31	2.39	3B_585846704	3B_592694374	98.9–110.5	585.846–592.694
*Qhd.saw-3B.2*	E1	2.67	0.44	3.18	3B_592694374	3B_508486802	110.5–119.8	592.694–508.486
*Qhd.saw-4A.1*	E3\E6	3.48–4.09	0.51–0.54	4.56–5.65	4A_700964251	4A_644613024	39.1–58.9	700.964–644.613
*Qhd.saw-4A.2*	E3\E6	3.71–4.47	0.54–0.57	5.63–5.67	4A_644613024	4A_672508553	58.9–73.9	644.613–672.508
*Qhd.saw-4A.3*	BLUP	5.73	0.49	6.12	4A_672508567	4A_672508702	79.2–79.8	672.508–672.508
*Qhd.saw-4A.4*	E1\E2\E5\E6\BLUP	3.08–8.38	0.53–0.84	4.73–12.04	4A_676486292	4A_638262359	86.3–96.7	676.486–638.262
*Qhd.saw-4A.5*	E1\E2\E5\BLUP	2.84–6.59	0.48–0.73	4.42–9.13	4A_638262359	4A_635663690	97.8–104.2	638.262–635.663
*Qhd.saw-4A.6*	E6	4.32	0.56	6.03	4A_639942192	4A_620278986	99.9–113.2	639.942–620.278
*Qhd.saw-4A.7*	E5	2.93	0.55	4.95	4A_604578311	4A_572876583	156.9–176.9	604.578–572.876
*Qhd.saw-4B.1*	E1\E3\BLUP	2.78–4.55	−0.46–−0.42	3.54–4.58	4B_494243528	4B_570190409	50.7–58.9	494.243–570.190
*Qhd.saw-4B.2*	BLUP	4.35	−0.41	4.40	4B_570190409	4B_618027704	58.9–65.7	570.190–618.027
*Qhd.saw-4B.3*	E3	2.64	−0.45	3.45	4B_621814323	4B_642460586	67.5–75.5	621.814–642.460
*Qhd.saw-4D.1*	E4	2.52	−0.69	4.22	4D_481390025	4D_433505595	0.0–9.1	481.390–433.505
*Qhd.saw-4D.2*	E2	6.57	−0.84	9.74	4D_48697668	4D_28547729	35.9–38.9	48.697–28.547
*Qhd.saw-4D.3*	E1\E2\E3\E4\E5\BLUP	4.46–22.14	−1.32–−0.97	8.35–29.52	4D_28547729	4D_15772687	39.6–46.9	28.547–15.772
*Qhd.saw-5A.1*	E1	2.55	−0.44	3.26	5A_462122669	5A_467547080	190.4–193.4	462.122–467.547
*Qhd.saw-5A.2*	E1\E3\E6	3.12–4.31	−0.58–−0.48	3.96–5.70	5A_467547080	5A_445264090	193.6–209.5	467.547–445.264
*Qhd.saw-5A.3*	E3	3.75	−0.55	5.27	5A_445264090	5A_445832627	211.3–214.4	445.264–445.832
*Qhd.saw-5A.4*	BLUP	5.05	−0.46	5.33	5A_395317374	5A_47016091	236.6–245.9	395.317–47.016
*Qhd.saw-5D.1*	E2\E6\BLUP	2.77–3.10	−0.54–−0.32	2.58–4.26	5D_390409478	5D_370312603	0.0–7.0	390.409–370.312
*Qhd.saw-5D.2*	E2\E6\BLUP	3.24–3.77	−0.58–−0.35	3.20–4.83	5D_370312603	5D_259452342	7.0–20.2	370.312–259.452
*Qhd.saw-5D.3*	E4\BLUP	2.81–3.07	−0.52–−0.34	2.90–4.10	5D_138877074	5D_34236785	26.2–33.5	138.877–34.236
*Qhd.saw-5D.4*	E5	3.19	−0.54	4.97	5D_47009022	5D_21550073	31.2–38.4	47.009–21.550
*Qhd.saw-7B.1*	E2	4.62	−0.66	6.07	7B_725766535	7B_725723913	0.0–2.5	725.766–725.723
*Qhd.saw-7B.2*	E2\BLUP	4.01–5.27	−0.70–−0.38	3.67–6.86	7B_718449045	7B_712710871	8.0–17.5	718.449–712.710

Note: Add., additive effect; and *R*^2^, percentage of explained variance.

**Table 3:** Additive effects of the five major QTLs for heading date (HD) in the DH population.

*Qhd.saw-3A.7*	*Qhd.saw-3A.8*	*Qhd.saw-3A.9*	*Qhd.saw-4A.4*	*Qhd.saw-4D.3*	Sample Size	HD (d)	Difference	Percent (%)
+	+	+	+	+	24	195.72 ± 1.06 a	4.58	2.40
−	+	−	+	+	1	195.66 ± 0.00 ab	4.53	2.37
−	+	+	+	+	1	195.51 ± 0.00 abc	4.37	2.29
−	−	+	+	+	1	195.20 ± 0.00 abcd	4.06	2.13
+	+	+	−	+	18	195.01 ± 1.52 abcd	3.88	2.03
−	−	+	−	−	1	194.89 ± 0.00 abcde	3.75	1.96
−	−	+	−	+	5	194.33 ± 0.64 bcde	3.19	1.67
−	−	−	+	+	22	194.29 ± 1.23 bcde	3.15	1.65
+	+	−	+	−	1	193.81 ± 0.00 bcdef	2.67	1.40
+	+	+	+	−	29	193.64 ± 1.54 bcdef	2.50	1.31
+	+	+	−	−	26	192.70 ± 1.28 cdef	1.56	0.82
−	−	−	−	+	16	192.50 ± 1.68 def	1.36	0.71
−	−	−	+	−	31	192.27 ± 1.69 ef	1.13	0.59
−	−	−	−	−	23	191.14 ± 1.20 f	0.00	0.00

Note: Values represent the mean heading date (HD) ± standard deviation. Letters indicate significant differences at *p* < 0.05. “+” and “−” denote lines with and without the favorable alleles of the target QTL based on the flanking markers of the corresponding QTL, respectively.

**Table 4:** Previously identified heading date (HD) QTLs on chromosomes 3A, 4A, and 4D.

Chr.	Left Marker	Right Marker	Physical Interval (Mb)	Reference
3A	wPt-4669	wPt-1665	279.47–432.91	Luo et al., 2016 [36]
3A	wPt-3041	wPt-4868	9.14–13.50	Rustgi et al., 2013 [37]
3A	IWB41929	-	714.43	Li et al., 2018 [38]
3A	IWB8499	-	638.45	Li et al., 2018 [38]
3A	IWB64668	-	176.56	Li et al., 2018 [38]
3A	IWA2738	-	13.90	Addison et al., 2016 [39]
3A	gwm247	-	197.43	Lee et al., 2014 [40]
3A	IWA7159	IWA4298	569.43–649.03	Mohler et al., 2016 [34]
3A	gwm133	-	509.54	Cuthbert et al., 2008 [41]
3A	mag1166	wPt-9369	14.85	Fan et al., 2019 [35]
3A	wmc264	-	625.79	Griffiths et al., 2009 [42]
3A	wmc532-wmc50	-	68.87	El-Feki et al., 2018 [43]
4A	barc1158	-	619.56	Sherman et al., 2014 [44]
4A	Jagger_c4331_105	BS00040647_51	606.40–594.66	Hu et al., 2020 [3]
4A	Kukri_c74409_199	-	37.77	Chen et al., 2020 [45]
4A	ACT/CAG-1	AAC/CAA-3	695.97	Nezhad et al., 2019 [46]
4A	IWA8209	IWA2761	615.71–626.06	Mohler et al., 2016 [34]
4A	AX-89555314	AX-89450319	2.95–6.04	Fan et al., 2019 [35]
4A	AX-95239105	AX-111711476	669.58	Fan et al., 2019 [35]
4D	wPt-0941	wPt-2379	52.63–196.64	Sherman et al., 2014 [44]

Note: If certain markers could not be accurately positioned on the physical map, their locations were estimated using the positions of adjacent markers. In cases where the positions of these adjacent markers were also indeterminate, the markers in question were treated as single-marker loci.

**Table 5:** Detailed information on highly expressed candidate genes for heading date.

QTL Name	Chr.	Gene	Physical Interval (Mb)	Rice Homologous Gene	Function Description
*Qhd.saw-3A.9*	3A	*TraesCS3A02G595800LC*	688.570216–688.570572	-	Calcium-dependent lipid-binding (CaLB domain) family protein
*Qhd.saw-3A.9*	3A	*TraesCS3A02G446400*	686.910184–686.911554	-	Kelch repeat-containing F-box protein-like
*Qhd.saw-3A.9*	3A	*TraesCS3A02G587100LC*	683.081967–683.085806	-	UDP-glycosyltransferase 84A1
*Qhd.saw-3A.9*	3A	*TraesCS3A02G440800*	683.399747–683.402388	*Os01t0926600-01*	glycosyltransferase family exostosin protein
*Qhd.saw-3A.9*	3A	*TraesCS3A02G440100*	683.335397–683.338550	*Os01t0926600-01*	glycosyltransferase family exostosin protein
*Qhd.saw-4D.3*	4D	*TraesCS4D02G038700*	17.057650–17.059578	*-*	Zinc finger family protein
*Qhd.saw-4D.3*	4D	*TraesCS4D02G046200*	22.217295–22.219478	*Os03t0711100-01*	CONSTANS-like zinc finger protein

## Data Availability

Data are contained within the article and Supplementary Materials.

## Supplementary Materials

The following supporting information can be downloaded at: https://www.mdpi.com/article/10.3390/genes15091150/s1, Table S1: Nutrient content of the soil under different phosphorus conditions; Table S2: Candidate genes identified within QTL regions of *Qhd.saw-3A.8*, *Qhd.saw-3A.9*, and *Qhd.saw-4D.3* for heading date in wheat.
