# Supplementary material for: Quantitative Trait Loci Mapping of Heading Date in Wheat under Phosphorus Stress Conditions
Source: Genes (Basel). 2024 Aug 31;15(9):1150. doi: 10.3390/genes15091150 (PMC11431698; doi:10.3390/genes15091150)
Supplement: Supplementary file 1 [file genes-15-01150-s001.zip › genes-3181128-supplementary.pdf]

Table S1. Nutrient content of the soil under different phosphorus conditions.

Treatment	normal P	medium P	low P
$CO(NH_2)_2$ ($kg \cdot ha^{-1}$)	168.75	168.75	168.75
P_2O_5 ($kg \cdot ha^{-1}$)	196	84	0
KCl ($kg \cdot ha^{-1}$)	49.17	49.17	49.17
N (%)	0.1	0.1	0.1
P ($mg \cdot kg^{-1}$)	10.9	6.1	3.47
K ($mg \cdot kg^{-1}$)	190	196	192
SOM ($g \cdot kg^{-1}$)	23.8	23.8	23.4
pH	8.21	8.25	8.17
EC ($\mu s \cdot cm^{-1}$)	125.3	120.2	130.8

Table S2. Candidate genes identified within QTL regions of Qhd.saw-3A.8, Qhd.saw-3A.9, and Qhd.saw-4D.3 for heading date in wheat.

Chr.	Start	End	Gene	Strand	Function description
chr3A	651.718441Mb	651.718551Mb	*TraesCS3A02G548200LC*	+	Cytochrome b6-f complex subunit 5
chr3A	651.780047Mb	651.783275Mb	*TraesCS3A02G406500*	+	PISTILLATA-like MADS-box transcription factor
chr3A	651.838228Mb	651.842348Mb	*TraesCS3A02G548800LC*	-	F-box domain containing protein, expressed
chr3A	653.008909Mb	653.013067Mb	*TraesCS3A02G407700*	+	F-box family protein
chr3A	654.021131Mb	654.021326Mb	*TraesCS3A02G550000LC*	+	hydroxyproline-rich glycoprotein family protein
chr3A	654.173041Mb	654.175948Mb	*TraesCS3A02G409500*	-	Glycosyl hydrolase family protein
chr3A	654.709256Mb	654.710511Mb	*TraesCS3A02G410400*	+	F-box family protein
chr3A	654.758357Mb	654.759672Mb	*TraesCS3A02G410500*	+	F-box protein-like
chr3A	654.773258Mb	654.77399Mb	*TraesCS3A02G551400LC*	-	F-box/LRR-repeat protein 25
chr3A	654.969679Mb	654.972859Mb	*TraesCS3A02G410700*	+	F-box protein-like
chr3A	655.268496Mb	655.27205Mb	*TraesCS3A02G411000*	+	F-box protein-like
chr3A	655.75314Mb	655.754279Mb	*TraesCS3A02G411300*	-	F-box family protein
chr3A	655.756894Mb	655.761352Mb	*TraesCS3A02G551800LC*	-	F-box family protein
chr3A	655.770153Mb	655.771334Mb	*TraesCS3A02G411400*	-	F-box protein family
chr3A	681.824856Mb	681.828496Mb	*TraesCS3A02G438700*	-	S-acyltransferase
chr3A	682.479467Mb	682.48052Mb	*TraesCS3A02G439100*	+	RING/FYVE/PHD zinc finger superfamily protein
chr3A	682.754286Mb	682.754627Mb	*TraesCS3A02G587000LC*	-	zinc finger MYM-type-like protein
chr3A	683.081967Mb	683.085806Mb	*TraesCS3A02G587100LC*	+	UDP-glycosyltransferase 84A1
chr3A	683.138534Mb	683.142369Mb	*TraesCS3A02G439900*	-	glycosyltransferase family exostosin protein
chr3A	683.335397Mb	683.33855Mb	*TraesCS3A02G440100*	-	glycosyltransferase family exostosin protein
chr3A	683.399747Mb	683.402388Mb	*TraesCS3A02G440800*	-	glycosyltransferase family exostosin protein
chr3A	684.898189Mb	684.898419Mb	*TraesCS3A02G589300LC*	-	TTF-type zinc finger protein with HAT dimerization domain
chr3A	684.898447Mb	684.899021Mb	*TraesCS3A02G589400LC*	-	zinc finger MYM-type-like protein
chr3A	686.312444Mb	686.315651Mb	*TraesCS3A02G445100*	+	F-box family protein
chr3A	686.319165Mb	686.323743Mb	*TraesCS3A02G590800LC*	+	F-box family protein

chr3A	686.653103Mb	686.653675Mb	*TraesCS3A02G591300LC*	-	zinc finger nuclease 2
chr3A	686.6582Mb	686.662687Mb	*TraesCS3A02G445700*	-	F-box family protein
chr3A	686.677237Mb	686.679602Mb	*TraesCS3A02G445800*	-	F-box family protein
chr3A	686.874693Mb	686.875933Mb	*TraesCS3A02G446100*	-	F-box family protein
chr3A	686.910184Mb	686.911554Mb	*TraesCS3A02G446400*	-	Kelch repeat-containing F-box protein-like
chr3A	687.60319Mb	687.604778Mb	*TraesCS3A02G447400*	+	F-box family protein
chr3A	687.622382Mb	687.622975Mb	*TraesCS3A02G593300LC*	-	UDP-Glycosyltransferase superfamily protein
chr3A	687.623081Mb	687.623584Mb	*TraesCS3A02G593400LC*	-	F-box domain containing protein, expressed
chr3A	687.630122Mb	687.630577Mb	*TraesCS3A02G593600LC*	-	F-box family protein
chr3A	687.764985Mb	687.76517Mb	*TraesCS3A02G593900LC*	-	F-box family protein
chr3A	688.3107Mb	688.31099Mb	*TraesCS3A02G448000*	+	Zinc finger homeodomain protein
chr3A	688.343319Mb	688.343609Mb	*TraesCS3A02G448100*	+	Zinc finger homeodomain protein
chr3A	688.560574Mb	688.561056Mb	*TraesCS3A02G595600LC*	-	F-box protein
chr3A	688.570216Mb	688.570572Mb	*TraesCS3A02G595800LC*	+	Calcium-dependent lipid-binding (CaLB domain) family protein
chr4D	15.881546Mb	15.883496Mb	*TraesCS4D02G033800*	-	Cytokinin riboside 5'-monophosphate phosphoribohydrolase
chr4D	15.949391Mb	15.950521Mb	*TraesCS4D02G033900*	+	Zinc finger family protein
chr4D	16.142095Mb	16.144347Mb	*TraesCS4D02G034300*	+	Zinc finger CCCH domain-containing protein
chr4D	16.357932Mb	16.360052Mb	*TraesCS4D02G035300*	-	F-box protein
chr4D	16.790537Mb	16.792672Mb	*TraesCS4D02G037800*	-	Glycosyltransferase
chr4D	16.793676Mb	16.795542Mb	*TraesCS4D02G037900*	-	UDP-glycosyltransferase
chr4D	17.05765Mb	17.059578Mb	*TraesCS4D02G038700*	-	Zinc finger family protein
chr4D	17.905217Mb	17.906494Mb	*TraesCS4D02G039800*	+	F-box protein
chr4D	19.710052Mb	19.710258Mb	*TraesCS4D02G027800LC*	+	F-box/RNI-like superfamily protein
chr4D	19.745126Mb	19.746578Mb	*TraesCS4D02G041000*	+	F-box family protein
chr4D	19.808217Mb	19.809798Mb	*TraesCS4D02G041300*	+	Cytochrome P450
chr4D	19.856418Mb	19.859552Mb	*TraesCS4D02G028100LC*	+	zinc finger MYM-type-like protein
chr4D	19.9518Mb	19.957109Mb	*TraesCS4D02G041700*	+	Zinc finger protein VAR3, chloroplastic
chr4D	20.277209Mb	20.27742Mb	*TraesCS4D02G028700LC*	+	Zinc finger CCCH domain-containing protein 13
chr4D	20.308237Mb	20.309031Mb	*TraesCS4D02G030700LC*	-	Photosystem I P700 chlorophyll a apoprotein A2
chr4D	20.309774Mb	20.310412Mb	*TraesCS4D02G030800LC*	-	Photosystem I P700 chlorophyll a apoprotein A2
chr4D	20.310589Mb	20.31144Mb	*TraesCS4D02G030900LC*	-	Photosystem I P700 chlorophyll a apoprotein A1
chr4D	20.312229Mb	20.312716Mb	*TraesCS4D02G031000LC*	-	Photosystem I P700 chlorophyll a apoprotein A1
chr4D	20.314697Mb	20.314954Mb	*TraesCS4D02G031100LC*	+	Photosystem I reaction center subunit IX
chr4D	20.318846Mb	20.319439Mb	*TraesCS4D02G031500LC*	+	Photosystem II CP47 reaction center protein
chr4D	20.319535Mb	20.319903Mb	*TraesCS4D02G031600LC*	+	Photosystem II CP47 reaction center protein
chr4D	20.319919Mb	20.320128Mb	*TraesCS4D02G031700LC*	+	Photosystem II CP47 reaction center protein
chr4D	20.320252Mb	20.320371Mb	*TraesCS4D02G031800LC*	+	Photosystem II CP47 reaction center protein
chr4D	20.320933Mb	20.321154Mb	*TraesCS4D02G043300*	+	Photosystem II reaction center protein H
chr4D	21.600242Mb	21.602055Mb	*TraesCS4D02G036200LC*	-	zinc finger MYM-type-like protein
chr4D	22.217295Mb	22.219478Mb	*TraesCS4D02G046200*	-	CONSTANS-like zinc finger protein
chr4D	22.472713Mb	22.474934Mb	*TraesCS4D02G046500*	+	Zinc-finger domain of monoamine-oxidase A repressor R1, putative
chr4D	22.477284Mb	22.481086Mb	*TraesCS4D02G046600*	+	Zinc finger protein VAR3, chloroplastic
chr4D	23.924802Mb	23.925137Mb	*TraesCS4D02G038300LC*	-	cytochrome P450, family 711, subfamily A, polypeptide 1
chr4D	24.400337Mb	24.400642Mb	*TraesCS4D02G038800LC*	+	zinc finger WD40 repeat protein 1
